# Supplementary material for: Polyketide synthase gene clusters in the ericoid mycorrhizal fungus Oidiodendron maius: genome-wide prediction, expression profiling, and functional characterization of knockout mutants
Source: Front Plant Sci. 2026 Sep 16;17:1924430. doi: 10.3389/fpls.2026.1924430 (PMC13623903; doi:10.3389/fpls.2026.1924430)
Supplement: Supplementary file 1 [file SupplementaryFile1.zip › Supplementary Materials/Supplementary Folder 1.zip!/Oidma1_AssemblyScaffolds_Repeatmasked/knownclusterblast/cluster20/jgi.p_Oidma1_164694_mibig_hits.html]

| MIBiG Protein | Description | MIBiG Cluster | MiBiG Product | % ID | % Coverage | BLAST Score | E-value |
| --- | --- | --- | --- | --- | --- | --- | --- |
| EFY94505.1 | multidrug\_resistance\_protein | BGC0000337 | Destruxins\_biosynthetic\_gene\_cluster | 35 | 98.4848484848 | 652 | 1.2e-186 |
| AAS92552.1 | SirA | BGC0001044 | Sirodesmin\_biosynthetic\_gene\_cluster | 33 | 100.398724083 | 616 | 7.1e-176 |
| AET14838.1 | multidrug\_resistance\_protein | BGC0001274\_c2 | Non-acetylated\_open-chain\_sophorolipid\_/\_diacetylated\_lactonic | 34 | 101.435406699 | 613 | 6e-175 |
| ctg1\_orf5 |  | BGC0000321 | Chaetocin\_biosynthetic\_gene\_cluster | 32 | 92.7432216906 | 462 | 2.2e-129 |
| XP\_011392729.1 | ABC\_transporter | BGC0001281 | Ustilagic\_acid\_biosynthetic\_gene\_cluster | 35 | 43.5406698565 | 305 | 3.5e-82 |
| AIS24845.1 | Dst21 | BGC0001147 | Distamycin\_biosynthetic\_gene\_cluster | 29 | 45.2950558214 | 215 | 3.6e-55 |
| AGL93176.1 | ABC\_transporter\_ATP-binding\_protein | BGC0000560 | Subtilomycin\_biosynthetic\_gene\_cluster | 32 | 36.0446570973 | 213 | 1.4e-54 |
| BAE56595.1 |  | BGC0001123 | Aspirochlorine\_biosynthetic\_gene\_cluster | 30 | 46.4912280702 | 211 | 5.2e-54 |
| AAX31531.1 | putative\_ABC\_transporter | BGC0000336 | Daptomycin\_biosynthetic\_gene\_cluster | 29 | 46.2519936204 | 205 | 2.9e-52 |
| ABV83218.1 | CppG | BGC0000116 | Nystatin-like\_Pseudonocardia\_polyene\_biosynthetic\_gene\_cluster | 33 | 36.68261563 | 205 | 3.8e-52 |
| AGM16415.1 | paenibacterin\_ABC-transporter\_D | BGC0000400 | Paenibacterin\_biosynthetic\_gene\_cluster | 37 | 26.7145135566 | 202 | 3.2e-51 |
| AFJ14796.1 | PlpG | BGC0000403 | Pelgipeptin\_biosynthetic\_gene\_cluster | 37 | 26.63476874 | 202 | 3.2e-51 |
| CAJ88629.1 | putative\_netropsin\_resistance\_protein\_subunit\_2 | BGC0000327 | Congocidine\_biosynthetic\_gene\_cluster | 28 | 45.0558213716 | 202 | 3.2e-51 |
| AEZ51518.1 | PmxC | BGC0001153 | Polymyxin\_biosynthetic\_gene\_cluster | 37 | 26.9537480064 | 201 | 5.4e-51 |
| ACA97578.1 | PmxC | BGC0000408 | Polymyxin\_biosynthetic\_gene\_cluster | 37 | 26.9537480064 | 201 | 5.4e-51 |
| BAM09402.1 | putative\_ABC-transporter | BGC0000619 | Gassericin\_T\_biosynthetic\_gene\_cluster | 30 | 39.0749601276 | 200 | 9.2e-51 |
| ALX37944.1 | GaeT | BGC0001388 | Gassericin\_E\_biosynthetic\_gene\_cluster | 30 | 39.0749601276 | 200 | 1.2e-50 |
| CAG34715.1 | putative\_ABC\_transporter | BGC0000713 | Ribostamycin\_biosynthetic\_gene\_cluster | 31 | 43.4609250399 | 200 | 1.2e-50 |
| BAI23328.1 | putative\_ABC-transporter | BGC0000949 | A-500359s\_biosynthetic\_gene\_cluster | 31 | 40.9090909091 | 200 | 1.2e-50 |
| CAF33315.1 | putative\_ABC\_transporter,\_ATP-binding\_protein,\_NeoU | BGC0000709 | Neomycin\_biosynthetic\_gene\_cluster | 31 | 43.7799043062 | 198 | 3.5e-50 |
| CAH05098.1 | ABC\_transport\_protein | BGC0000710 | Neomycin\_biosynthetic\_gene\_cluster | 31 | 43.7799043062 | 198 | 3.5e-50 |
| CAH58693.1 | putative\_exporter | BGC0000711 | Neomycin\_biosynthetic\_gene\_cluster | 31 | 43.7799043062 | 198 | 3.5e-50 |
| AEH59056.1 | YvcC/ABC\_transporter | BGC0000385 | Lysobactin\_biosynthetic\_gene\_cluster | 30 | 46.1722488038 | 197 | 1e-49 |
| AFS60105.1 | PaenT | BGC0000540 | Paenibacillin\_biosynthetic\_gene\_cluster | 28 | 43.620414673 | 196 | 1.7e-49 |
| CAA12529.1 | ExsA\_protein | BGC0000802 | Succinoglycan\_biosynthetic\_gene\_cluster | 31 | 37.4003189793 | 195 | 3.9e-49 |
| AFJ14797.1 | PlpH | BGC0000403 | Pelgipeptin\_biosynthetic\_gene\_cluster | 42 | 20.7336523126 | 194 | 6.6e-49 |
| BAH43767.1 | ABC\_transporter\_ATP\_binding/permease\_protein | BGC0000452 | Tyrocidine\_biosynthetic\_gene\_cluster | 32 | 35.8851674641 | 194 | 8.6e-49 |
| ALK27913.1 | putative\_ABC\_transporter | BGC0001233 | Feglymycin\_biosynthetic\_gene\_cluster | 27 | 48.0861244019 | 194 | 8.6e-49 |
| AEH59102.1 | ABC\_transporter/permease/ATP-binding\_protein | BGC0000385 | Lysobactin\_biosynthetic\_gene\_cluster | 31 | 44.2583732057 | 194 | 8.6e-49 |
| AAM54082.1 | ABC\_transporter | BGC0000020 | Ansamitocin\_biosynthetic\_gene\_cluster | 30 | 40.5103668262 | 194 | 8.6e-49 |
| AHZ61877.1 | Lom43 | BGC0000240 | Lomaiviticin\_biosynthetic\_gene\_cluster | 30 | 37.8787878788 | 191 | 4.3e-48 |
| CAA11793.1 | PCZA363.2 | BGC0000322 | Chloroeremomycin\_biosynthetic\_gene\_cluster | 29 | 45.7735247209 | 191 | 5.6e-48 |
| ABP54659.1 | ABC\_transporter\_related | BGC0000241 | Lomaiviticin\_biosynthetic\_gene\_cluster | 32 | 39.0749601276 | 190 | 9.6e-48 |
| CAI47637.1 | putative\_ABC-type\_aminoglycoside\_exporter | BGC0000692 | Apramycin\_biosynthetic\_gene\_cluster | 28 | 43.7799043062 | 190 | 1.6e-47 |
| CAF32377.1 | putative\_ABC\_transporter,\_ATP-binding\_protein | BGC0000712 | Paromomycin\_biosynthetic\_gene\_cluster | 31 | 43.7001594896 | 189 | 2.1e-47 |
| AJM89736.1 | PmxC | BGC0001192 | Colistin\_biosynthetic\_gene\_cluster | 36 | 26.9537480064 | 189 | 2.1e-47 |
| AAC44070.1 | unknown | BGC0000797 | Sphingan\_polysaccharide\_biosynthetic\_gene\_cluster | 32 | 35.326953748 | 189 | 2.8e-47 |
| CAG38700.1 | putative\_ABC-transporter,\_ATP-binding\_protein | BGC0000708 | Lividomycin\_biosynthetic\_gene\_cluster | 31 | 41.5470494418 | 187 | 8.1e-47 |
| AAF71763.1 | NysG | BGC0000115 | Nystatin\_biosynthetic\_gene\_cluster | 29 | 37.9585326954 | 187 | 8.1e-47 |
| ABW74893.1 | ABC\_transporter | BGC0000759 | Diutan\_polysaccharide\_biosynthetic\_gene\_cluster | 32 | 35.7256778309 | 187 | 1.1e-46 |
| AAP57696.1 | putative\_secretion\_protein | BGC0000768 | Gellan\_polysaccharide\_biosynthetic\_gene\_cluster | 32 | 35.326953748 | 186 | 1.4e-46 |
| AFN69431.1 | ElxT | BGC0000509 | Epilancin\_15X\_biosynthetic\_gene\_cluster | 26 | 46.0127591707 | 186 | 2.4e-46 |
| AGS77319.1 | ABC\_transporter | BGC0001178 | UK-68,597\_biosynthetic\_gene\_cluster | 29 | 44.8963317384 | 185 | 3.1e-46 |
| BAJ19059.1 | putative\_ABC\_transporter | BGC0000288 | A-503083\_biosynthetic\_gene\_cluster | 29 | 41.1483253589 | 185 | 4e-46 |
| CAD91219.1 | putative\_ABC\_transporter,\_Dbv24 | BGC0000289 | A40926\_biosynthetic\_gene\_cluster | 32 | 31.1004784689 | 185 | 5.2e-46 |
| CAD89782.1 | hypothetical\_protein | BGC0001009 | Melithiazol\_biosynthetic\_gene\_cluster | 28 | 42.9027113238 | 184 | 6.9e-46 |
| ABS50188.1 | putative\_ABC\_transporter\_permease/ATP-binding\_protein | BGC0000410 | Pseudomonine\_biosynthetic\_gene\_cluster | 29 | 39.1547049442 | 184 | 9e-46 |
| AJF34465.1 | ABC\_transporter | BGC0001207 | Teixobactin\_biosynthetic\_gene\_cluster | 29 | 42.2647527911 | 184 | 9e-46 |
| AEP18658.1 | efflux\_transporter | BGC0000461 | WAP-8294A2\_(lotilibcin)\_biosynthetic\_gene\_cluster | 30 | 41.3875598086 | 183 | 1.2e-45 |
| CAC20925.1 | PimB\_protein | BGC0000125 | Pimaricin\_biosynthetic\_gene\_cluster | 28 | 48.3253588517 | 183 | 1.5e-45 |
| AIS24846.1 | Dst20 | BGC0001147 | Distamycin\_biosynthetic\_gene\_cluster | 32 | 31.8181818182 | 183 | 2e-45 |
| EFI65097.1 | lantibiotic\_transport\_protein | BGC0001407 | Bicereucin\_biosynthetic\_gene\_cluster | 31 | 36.3636363636 | 183 | 2e-45 |
| BAH43768.1 | ABC\_transporter\_ATP-binding/permease\_protein | BGC0000452 | Tyrocidine\_biosynthetic\_gene\_cluster | 28 | 35.7256778309 | 182 | 2.6e-45 |
| AAK81823.1 | ABC\_transporter | BGC0000326 | Complestatin\_biosynthetic\_gene\_cluster | 27 | 47.9266347687 | 181 | 4.4e-45 |
| CAG25756.1 | putative\_ABC\_transporter\_ATP-binding\_protein | BGC0000311 | Balhimycin\_biosynthetic\_gene\_cluster | 27 | 48.1658692185 | 181 | 4.4e-45 |
| CAG38699.1 | putative\_ABC-transporter,\_ATP-binding\_protein | BGC0000708 | Lividomycin\_biosynthetic\_gene\_cluster | 30 | 37.0813397129 | 181 | 5.8e-45 |
| WP\_006126653.1 | ABC\_transporter | BGC0000554 | SRO15-3108\_biosynthetic\_gene\_cluster | 29 | 46.9696969697 | 181 | 7.6e-45 |
| AAM80540.1 | StaU | BGC0000290 | A47934\_biosynthetic\_gene\_cluster | 29 | 39.6331738437 | 180 | 9.9e-45 |
| AEI58864.1 | ABC\_transporter | BGC0000455 | Vancomycin\_biosynthetic\_gene\_cluster | 29 | 47.6076555024 | 180 | 1.7e-44 |
| ADX66465.1 | ScnB | BGC0000108 | Natamycin\_biosynthetic\_gene\_cluster | 27 | 48.4848484848 | 179 | 2.9e-44 |
| ABQ84449.1 | salivaricin\_ABC-transporter | BGC0000624 | Salivaricin\_CRL1328\_alpha\_peptide\_/\_salivaricin\_CRL1328\_beta\_peptide | 28 | 47.2089314195 | 178 | 6.4e-44 |
| CAD15511.1 | probable\_composite\_atp-binding\_transmembrane\_abc\_transporter\_protein | BGC0001014 | Micacocidin\_biosynthetic\_gene\_cluster | 28 | 38.4370015949 | 178 | 6.4e-44 |
| CAP20371.1 | ABC\_transporter\_ATP-binding\_membrane\_protein | BGC0000347 | Equibactin\_biosynthetic\_gene\_cluster | 28 | 48.1658692185 | 178 | 6.4e-44 |
| CAP20370.1 | ABC\_transporter,\_ATP-binding\_membrane\_protein | BGC0000347 | Equibactin\_biosynthetic\_gene\_cluster | 30 | 35.8054226475 | 177 | 8.4e-44 |
| CAC17506.1 | putative\_ABC\_transporter\_ATP-binding\_protein | BGC0000324 | Coelibactin\_biosynthetic\_gene\_cluster | 32 | 38.5167464115 | 177 | 1.1e-43 |
| CAF32376.1 | putative\_ABC\_transporter,\_ATP-binding\_protein | BGC0000712 | Paromomycin\_biosynthetic\_gene\_cluster | 30 | 37.0015948963 | 176 | 2.4e-43 |
| AMM63163.1 | AniL | BGC0001371 | Echinocandin\_B\_biosynthetic\_gene\_cluster | 26 | 71.2121212121 | 176 | 2.4e-43 |
| AFD30963.1 | CrmT2 | BGC0000966 | Caerulomycin\_A\_biosynthetic\_gene\_cluster | 29 | 40.5103668262 | 175 | 3.2e-43 |
| EYT83450.1 | ABC\_transporter | BGC0001213 | Nataxazole\_biosynthetic\_gene\_cluster | 29 | 36.1244019139 | 175 | 3.2e-43 |
| AAK73498.1 | AmphG | BGC0000015 | Amphotericin\_biosynthetic\_gene\_cluster | 28 | 47.8468899522 | 174 | 9.3e-43 |
| CAE53357.1 | ABC\_transporter | BGC0000440 | Teicoplanin\_biosynthetic\_gene\_cluster | 28 | 49.2025518341 | 173 | 1.2e-42 |
| CAG15016.1 | ABC\_transporter | BGC0000441 | Teicoplanin\_biosynthetic\_gene\_cluster | 28 | 49.2025518341 | 173 | 1.2e-42 |
| ANP43732.1 | ThsT | BGC0001391 | Thusin\_biosynthetic\_gene\_cluster | 39 | 20.2551834131 | 173 | 1.6e-42 |
| BAJ19058.1 | putative\_ABC\_transporter | BGC0000288 | A-503083\_biosynthetic\_gene\_cluster | 29 | 39.3939393939 | 173 | 1.6e-42 |
| AHJ59536.1 | ABC\_transporter\_protein | BGC0000502 | Cerecidin\_/\_cerecidin\_A1\_/\_cerecidin\_A2\_/\_cerecidin\_A3\_/\_cerecidin | 31 | 34.1307814992 | 173 | 1.6e-42 |
| CAJ88628.1 | putative\_netropsin\_resistance\_protein\_subunit\_1 | BGC0000327 | Congocidine\_biosynthetic\_gene\_cluster | 29 | 48.0861244019 | 173 | 2.1e-42 |
| CAI47638.1 | putative\_ABC-type\_aminoglycoside\_exporter | BGC0000692 | Apramycin\_biosynthetic\_gene\_cluster | 30 | 31.8181818182 | 172 | 2.7e-42 |
| CAA90021.1 | PepT | BGC0000543 | Pep5\_biosynthetic\_gene\_cluster | 25 | 46.0127591707 | 172 | 3.5e-42 |
| BAB04170.1 | lantibiotic\_mersacidin\_transporter\_system | BGC0000517 | Haloduracin\_alpha\_/\_haloduracin\_beta\_biosynthetic\_gene\_cluster | 31 | 35.0079744817 | 171 | 4.6e-42 |
| CAJ87588.1 | putative\_inner\_membrane\_ABC-transporter | BGC0001055 | Yersiniabactin\_biosynthetic\_gene\_cluster | 30 | 40.9888357257 | 171 | 6e-42 |
| KIH99790.1 | hypothetical\_protein | BGC0001176 | Streptomonomicin\_biosynthetic\_gene\_cluster | 28 | 45.7735247209 | 171 | 6e-42 |
| CAG34716.1 | putative\_ABC\_transporter | BGC0000713 | Ribostamycin\_biosynthetic\_gene\_cluster | 29 | 38.1977671451 | 171 | 6e-42 |
| AFD30962.1 | CrmT3 | BGC0000966 | Caerulomycin\_A\_biosynthetic\_gene\_cluster | 29 | 42.3444976077 | 170 | 1e-41 |
| ALV82355.1 | ABC\_transporter | BGC0001370 | Lipopeptide\_8D1-1\_/\_lipopeptide\_8D1-2\_biosynthetic\_gene\_cluster | 29 | 43.3811802233 | 170 | 1.7e-41 |
| CAB38879.1 | putative\_ABC\_transporter | BGC0000315 | Calcium-dependent\_antibiotic\_biosynthetic\_gene\_cluster | 29 | 44.0988835726 | 169 | 2.3e-41 |
| BAE07080.1 | possible\_ABC\_transporter | BGC0000693 | Butirosin\_biosynthetic\_gene\_cluster | 27 | 43.620414673 | 169 | 3e-41 |
| BAL15734.1 | ABC\_transporter | BGC0000432 | Streptothricin\_biosynthetic\_gene\_cluster | 26 | 46.2519936204 | 168 | 3.9e-41 |
| CAD15510.1 | probable\_composite\_atp-binding\_transmembrane\_abc\_transporter\_protein | BGC0001014 | Micacocidin\_biosynthetic\_gene\_cluster | 42 | 20.1754385965 | 168 | 3.9e-41 |
| ABS50189.1 | putative\_ABC\_transporter\_permease/ATP-binding\_protein | BGC0000410 | Pseudomonine\_biosynthetic\_gene\_cluster | 27 | 38.995215311 | 168 | 3.9e-41 |
| CAJ87587.1 | putative\_ABC\_transporter\_protein | BGC0001055 | Yersiniabactin\_biosynthetic\_gene\_cluster | 27 | 44.8165869219 | 168 | 3.9e-41 |
| AFT91383.1 | EcdL | BGC0000338 | Echinocandin\_B\_biosynthetic\_gene\_cluster | 25 | 72.2488038278 | 168 | 3.9e-41 |
| CCC55904.1 | putative\_ABC\_transporter,\_ATPase\_and\_permease\_component | BGC0000973 | Collismycin\_A\_biosynthetic\_gene\_cluster | 28 | 43.3014354067 | 168 | 6.6e-41 |
| CAF31838.1 | putative\_hygromycin\_B\_exporter | BGC0000699 | Hygromycin\_B\_biosynthetic\_gene\_cluster | 28 | 35.8851674641 | 167 | 8.7e-41 |
| ABV83219.1 | CppH | BGC0000116 | Nystatin-like\_Pseudonocardia\_polyene\_biosynthetic\_gene\_cluster | 27 | 37.8787878788 | 167 | 1.1e-40 |
| AAF71764.1 | NysH | BGC0000115 | Nystatin\_biosynthetic\_gene\_cluster | 27 | 42.1052631579 | 166 | 1.5e-40 |
| BAI23327.1 | putative\_ABC-transporter | BGC0000949 | A-500359s\_biosynthetic\_gene\_cluster | 29 | 37.1610845295 | 166 | 1.5e-40 |
| CAC17507.1 | putative\_ABC\_transporter\_ATP-binding\_protein | BGC0000324 | Coelibactin\_biosynthetic\_gene\_cluster | 28 | 39.7129186603 | 166 | 1.5e-40 |
| EDY04298.1 | ABC\_transporter-related\_protein | BGC0000516 | Geobacillin\_II\_biosynthetic\_gene\_cluster | 30 | 36.8421052632 | 166 | 2.5e-40 |
| ADI10107.1 | cytolysin\_B\_transport\_protein | BGC0001229 | SBI\_06990\_alpha\_/\_SBI\_06989\_beta\_biosynthetic\_gene\_cluster | 34 | 26.5550239234 | 166 | 2.5e-40 |
| AAG32538.1 | SalT | BGC0000548 | Salivaricin\_A\_biosynthetic\_gene\_cluster | 35 | 20.653907496 | 165 | 3.3e-40 |
| ALE27499.1 | ABC\_transporter | BGC0001292 | A33853\_biosynthetic\_gene\_cluster | 29 | 38.9154704944 | 165 | 5.6e-40 |
| EEM14530.1 | hypothetical\_protein | BGC0001210 | Pseudomycoicidin\_biosynthetic\_gene\_cluster | 29 | 47.288676236 | 165 | 5.6e-40 |
| EYT83451.1 | ABC\_transporter\_ATP-binding\_protein | BGC0001213 | Nataxazole\_biosynthetic\_gene\_cluster | 30 | 35.8054226475 | 164 | 7.3e-40 |
| CAM02315.1 | putative\_ABC\_transporter\_transmembrane | BGC0000349 | Erythrochelin\_biosynthetic\_gene\_cluster | 27 | 47.9266347687 | 163 | 1.3e-39 |
| WP\_010071619.1 | ABC\_transporter\_ATP-binding\_protein | BGC0000578 | SRO15-2005\_biosynthetic\_gene\_cluster | 27 | 46.331738437 | 163 | 1.6e-39 |
| AAY93395.1 | 2-phenylethylamine\_uptake\_protein\_PeaH | BGC0000413 | Pyoverdine\_biosynthetic\_gene\_cluster | 30 | 35.0877192982 | 163 | 2.1e-39 |
| CCC55903.1 | putative\_ABC\_transporter,\_ATPase\_and\_permease\_component | BGC0000973 | Collismycin\_A\_biosynthetic\_gene\_cluster | 28 | 42.0255183413 | 161 | 6.2e-39 |
| ABL74935.1 | TlmT | BGC0001048 | Tallysomycin\_biosynthetic\_gene\_cluster | 29 | 42.663476874 | 161 | 8.1e-39 |
| CAC20924.1 | PimA\_protein | BGC0000125 | Pimaricin\_biosynthetic\_gene\_cluster | 26 | 41.3875598086 | 161 | 8.1e-39 |
| ALR96371.1 | lantibiotic\_ABC\_transporter | BGC0001289 | Penisin\_biosynthetic\_gene\_cluster | 28 | 37.3205741627 | 160 | 1.4e-38 |
| EHQ60564.1 | ABC\_transporter | BGC0001356 | Paeninodin\_biosynthetic\_gene\_cluster | 39 | 20.0956937799 | 158 | 4e-38 |
| AHF21240.1 | CrnT | BGC0000500 | Carnolysin\_A1\_/\_carnolysin\_A2\_biosynthetic\_gene\_cluster | 26 | 39.7926634769 | 158 | 5.3e-38 |
| ACU62760.1 | ABC\_transporter\_related | BGC0001392 | Pinensins\_biosynthetic\_gene\_cluster | 27 | 36.283891547 | 157 | 9e-38 |
| AFY23032.1 | TamA | BGC0000274 | Tetarimycin\_biosynthetic\_gene\_cluster | 33 | 23.0462519936 | 157 | 1.2e-37 |
| ACP43452.1 | McnB | BGC0000590 | Microcin\_N\_biosynthetic\_gene\_cluster | 31 | 33.971291866 | 156 | 2.6e-37 |
| ALE27500.1 | ABC\_transporter | BGC0001292 | A33853\_biosynthetic\_gene\_cluster | 27 | 43.7799043062 | 155 | 3.4e-37 |
| ADX66464.1 | ScnA | BGC0000108 | Natamycin\_biosynthetic\_gene\_cluster | 27 | 46.2519936204 | 155 | 4.5e-37 |
| AHH99931.1 | ABC\_transporter\_protein | BGC0000002 | Aculeximycin\_biosynthetic\_gene\_cluster | 27 | 40.350877193 | 154 | 7.6e-37 |
| BAD72775.1 |  | BGC0000552 | Smb\_A\_/\_Smb\_B\_biosynthetic\_gene\_cluster | 26 | 49.3620414673 | 153 | 2.2e-36 |
| AAK73499.1 | AmphH | BGC0000015 | Amphotericin\_biosynthetic\_gene\_cluster | 28 | 41.5470494418 | 152 | 2.9e-36 |
| ANQ31705.1 | ABC\_transporter | BGC0001395 | Teichomycin\_biosynthetic\_gene\_cluster | 34 | 25.3588516746 | 152 | 3.8e-36 |
| AAG28766.1 | CoaD | BGC0000617 | Coagulin\_biosynthetic\_gene\_cluster | 39 | 20.0956937799 | 151 | 4.9e-36 |
| BAL72550.1 | lariatin\_biosynthetic\_protein | BGC0000575 | Lariatin\_biosynthetic\_gene\_cluster | 28 | 38.3572567783 | 151 | 6.4e-36 |
| CAB60262.1 | MrsT\_protein | BGC0000527 | Mersacidin\_biosynthetic\_gene\_cluster | 27 | 40.7496012759 | 150 | 1.1e-35 |
| CAB53323.1 | putative\_ABC-transporter\_transmembrane\_protein | BGC0000325 | Coelichelin\_biosynthetic\_gene\_cluster | 27 | 44.976076555 | 146 | 2.1e-34 |
| BAG22745.1 | putative\_ABC-type\_multidrug\_transport\_system\_ATPase\_and\_permease\_component | BGC0000724 | Streptomycin\_biosynthetic\_gene\_cluster | 29 | 41.2280701754 | 145 | 3.5e-34 |
| CAH94306.1 | putative\_ABC\_transporter\_StrW | BGC0000717 | Streptomycin\_biosynthetic\_gene\_cluster | 29 | 41.2280701754 | 145 | 3.5e-34 |
| AAP03990.1 | putative\_microcin\_L\_transport\_protein | BGC0000588 | Microcin\_L\_biosynthetic\_gene\_cluster | 29 | 33.8915470494 | 145 | 3.5e-34 |
| WP\_043270371.1 | ABC\_transporter\_ATPase | BGC0001188 | Legonaridin\_biosynthetic\_gene\_cluster | 26 | 42.9824561404 | 144 | 7.9e-34 |
| AAK27372.1 | ABC\_transporter | BGC0000623 | Plantaricin\_W\_alpha\_/\_plantaricin\_W\_beta\_biosynthetic\_gene\_cluster | 26 | 39.2344497608 | 143 | 2.3e-33 |
| AHW81481.1 | hypothetical\_protein | BGC0000934 | Endophenazines\_biosynthetic\_gene\_cluster | 28 | 47.4481658692 | 142 | 3e-33 |
| AFW17836.1 | PaeT | BGC0000541 | Paenicidin\_A\_biosynthetic\_gene\_cluster | 25 | 36.7623604466 | 141 | 6.7e-33 |
| AAK01464.1 | ABC\_transporter | BGC0000412 | Pyochelin\_biosynthetic\_gene\_cluster | 33 | 22.5677830941 | 140 | 1.5e-32 |
| CAJ44959.1 | MchF\_protein | BGC0000587 | Microcin\_H47\_biosynthetic\_gene\_cluster | 28 | 34.2902711324 | 139 | 1.9e-32 |
| CAE55703.1 | probable\_microcin\_H47\_secretion\_ATP-binding\_protein\_mchF | BGC0000589 | Microcin\_M\_biosynthetic\_gene\_cluster | 28 | 34.2902711324 | 139 | 2.5e-32 |
| AAL08400.1 | MceG | BGC0000586 | Microcin\_E492\_biosynthetic\_gene\_cluster | 28 | 34.2105263158 | 137 | 9.6e-32 |
| AAK01462.1 | ABC\_transporter | BGC0000412 | Pyochelin\_biosynthetic\_gene\_cluster | 29 | 36.68261563 | 134 | 6.2e-31 |
| CAM03501.1 | putative\_ABC\_transporter | BGC0000513 | Erythreapeptin\_biosynthetic\_gene\_cluster | 26 | 45.4545454545 | 130 | 1.2e-29 |
| CAB01940.1 | ExpD1 | BGC0000801 | Galactoglucan\_biosynthetic\_gene\_cluster | 26 | 33.0940988836 | 129 | 2.6e-29 |
| BAU50927.1 | ABC\_transporter | BGC0001379 | BD-12\_biosynthetic\_gene\_cluster | 23 | 47.2089314195 | 128 | 7.6e-29 |
| ACU73106.1 | ABC\_transporter\_related | BGC0000501 | Catenulipeptin\_biosynthetic\_gene\_cluster | 28 | 36.3636363636 | 124 | 8.4e-28 |
| AAX31526.1 | putative\_ABC\_transporter | BGC0000336 | Daptomycin\_biosynthetic\_gene\_cluster | 25 | 48.8835725678 | 124 | 1.1e-27 |
| AHW81482.1 | hypothetical\_protein | BGC0000934 | Endophenazines\_biosynthetic\_gene\_cluster | 29 | 32.8548644338 | 123 | 1.4e-27 |
| CBG75488.1 | putative\_ABC\_transport\_system\_integral\_membrane\_component | BGC0000423 | Scabichelin\_biosynthetic\_gene\_cluster | 24 | 38.038277512 | 122 | 3.2e-27 |
| ADJ56354.1 | NisT | BGC0000535 | Nisin\_A\_biosynthetic\_gene\_cluster | 30 | 20.0956937799 | 121 | 9.3e-27 |
| CAA19962.1 | putative\_ABC\_transporter | BGC0000551 | SapB\_biosynthetic\_gene\_cluster | 26 | 39.6331738437 | 120 | 1.2e-26 |
| CAA79469.1 | NisT\_protein | BGC0000538 | Nisin\_Z\_biosynthetic\_gene\_cluster | 30 | 20.4146730463 | 120 | 1.6e-26 |
| AEX55160.1 | putative\_ABC\_transporter\_SlvT | BGC0000549 | Salivaricin\_D\_biosynthetic\_gene\_cluster | 26 | 36.5231259968 | 119 | 2.7e-26 |
| AAD56148.1 | MutT | BGC0000533 | Mutacin\_III\_biosynthetic\_gene\_cluster | 23 | 43.9393939394 | 119 | 2.7e-26 |
| WP\_020635024.1 | multidrug\_ABC\_transporter\_ATPase | BGC0001211 | Albachelin\_biosynthetic\_gene\_cluster | 23 | 38.6762360447 | 119 | 3.5e-26 |
| CAM02316.1 | ABC\_transporter\_protein,\_ATP-binding\_component | BGC0000349 | Erythrochelin\_biosynthetic\_gene\_cluster | 24 | 44.1786283892 | 118 | 6e-26 |
| EMC15128.1 | ABC\_transporter,\_ATP-binding\_and\_permease\_protein | BGC0000530 | Mutacin\_B-Ny266\_biosynthetic\_gene\_cluster | 23 | 43.9393939394 | 118 | 6e-26 |
| BAG19224.1 | membrane\_translocator | BGC0000496 | AmfS\_biosynthetic\_gene\_cluster | 28 | 40.350877193 | 117 | 1e-25 |
| ABA00880.1 | NsuT | BGC0000537 | Nisin\_U\_biosynthetic\_gene\_cluster | 31 | 20.653907496 | 113 | 1.5e-24 |
| AAN85531.1 | ABC\_transporter\_component,\_ATP\_hydrolase | BGC0001101 | Leinamycin\_biosynthetic\_gene\_cluster | 36 | 20.2551834131 | 113 | 1.5e-24 |
| AAX31527.1 | putative\_ABC\_transporter | BGC0000336 | Daptomycin\_biosynthetic\_gene\_cluster | 26 | 37.2408293461 | 111 | 7.4e-24 |
| AAC63532.1 | sublancin\_transporter\_protein | BGC0000558 | Sublancin\_168\_biosynthetic\_gene\_cluster | 23 | 45.6140350877 | 107 | 1.4e-22 |
| BAG22744.1 | putative\_ABC-type\_multidrug\_transport\_system\_ATPase\_and\_permease\_component | BGC0000724 | Streptomycin\_biosynthetic\_gene\_cluster | 24 | 36.2041467305 | 101 | 5.8e-21 |
| CAH94305.1 | putative\_ABC\_transporter\_StrV | BGC0000717 | Streptomycin\_biosynthetic\_gene\_cluster | 24 | 36.2041467305 | 101 | 5.8e-21 |
| EWM63070.1 | ABC\_transporter\_ATP-binding\_protein | BGC0000679 | Diazepinomicin\_biosynthetic\_gene\_cluster | 24 | 39.3141945774 | 100 | 1.3e-20 |
| AAD28497.1 | McjD | BGC0000581 | Microcin\_J25\_biosynthetic\_gene\_cluster | 25 | 35.1674641148 | 97 | 1.1e-19 |
| CAD91242.1 | hypothetical\_protein | BGC0000289 | A40926\_biosynthetic\_gene\_cluster | 31 | 20.0956937799 | 94 | 7.1e-19 |
| ADC45593.1 | ABC\_transporter | BGC0000093 | Meilingmycin\_biosynthetic\_gene\_cluster | 27 | 24.4816586922 | 94 | 1.2e-18 |
| BAG19223.1 | membrane\_translocator | BGC0000496 | AmfS\_biosynthetic\_gene\_cluster | 25 | 43.5406698565 | 92 | 4.6e-18 |
| AMR44284.1 | ABC\_transporter | BGC0001398 | Phomopsins\_biosynthetic\_gene\_cluster | 25 | 29.1068580542 | 91 | 1e-17 |
| BAC98761.2 | NukT | BGC0000539 | Nukacin\_ISK-1\_biosynthetic\_gene\_cluster | 27 | 21.2121212121 | 89 | 2.3e-17 |
| CAB53325.1 | putative\_iron-siderophore\_ABC-transporter\_ATP-binding\_protein | BGC0000325 | Coelichelin\_biosynthetic\_gene\_cluster | 25 | 20.4146730463 | 88 | 6.7e-17 |
| CAA07372.1 | StrV | BGC0000690 | 5'-hydroxystreptomycin\_biosynthetic\_gene\_cluster | 23 | 46.6507177033 | 84 | 7.4e-16 |
| ABI30230.1 | McdT | BGC0000526 | Macedocin\_biosynthetic\_gene\_cluster | 28 | 20.5741626794 | 84 | 1.3e-15 |
| ACB12564.1 | Fum19 | BGC0000063 | Fumonisin\_biosynthetic\_gene\_cluster | 27 | 24.4019138756 | 77 | 1.5e-13 |
| AAM70341.1 | CalT5 | BGC0000033 | Calicheamicin\_biosynthetic\_gene\_cluster | 27 | 20.0159489633 | 74 | 1.3e-12 |
| ABV79989.1 | ApnE | BGC0000301 | Anabaenopeptin\_biosynthetic\_gene\_cluster | 25 | 20.8133971292 | 58 | 5.7e-08 |
| OAQ83754.1 | ABC\_multidrug\_transporter | BGC0001358 | Leucinostatins\_biosynthetic\_gene\_cluster | 25 | 22.6475279107 | 58 | 7.4e-08 |
| AHZ61838.1 | Lom4 | BGC0000240 | Lomaiviticin\_biosynthetic\_gene\_cluster | 27 | 21.3716108453 | 54 | 1.4e-06 |
| ABP54626.1 | ABC\_transporter\_related | BGC0000241 | Lomaiviticin\_biosynthetic\_gene\_cluster | 25 | 21.6108452951 | 51 | 6.9e-06 |
